# Mechanisms Contributing to the Comorbidity of COPD and Lung Cancer

**DOI:** 10.3390/ijms24032859

**Published:** 2023-02-02

**Authors:** Aisling Forder, Rebecca Zhuang, Vanessa G. P. Souza, Liam J. Brockley, Michelle E. Pewarchuk, Nikita Telkar, Greg L. Stewart, Katya Benard, Erin A. Marshall, Patricia P. Reis, Wan L. Lam

**Affiliations:** 1 British Columbia Cancer Research Institute, Vancouver, BC V5Z 1L3, Canada; 2 Faculty of Medicine, University of British Columbia, Vancouver, BC V6T 1Z3, Canada; 3 Molecular Oncology Laboratory, Experimental Research Unit, School of Medicine, São Paulo State University (UNESP), Botucatu 18618-687, SP, Brazil; 4 Department of Medical Genetics, University of British Columbia, Vancouver, BC V6H 3N1, Canada; 5 British Columbia Children’s Hospital Research Institute, Vancouver, BC V5Z 4H4, Canada

**Keywords:** COPD, lung cancer, pathogenesis, microbiome, immune microenvironment, genomic alterations, epigenetics, lung cancer screening

## Abstract

Lung cancer and chronic obstructive pulmonary disease (COPD) often co-occur, and individuals with COPD are at a higher risk of developing lung cancer. While the underlying mechanism for this risk is not well understood, its major contributing factors have been proposed to include genomic, immune, and microenvironment dysregulation. Here, we review the evidence and significant studies that explore the mechanisms underlying the heightened lung cancer risk in people with COPD. Genetic and epigenetic changes, as well as the aberrant expression of non-coding RNAs, predispose the lung epithelium to carcinogenesis by altering the expression of cancer- and immune-related genes. Oxidative stress generated by tobacco smoking plays a role in reducing genomic integrity, promoting epithelial-mesenchymal-transition, and generating a chronic inflammatory environment. This leads to abnormal immune responses that promote cancer development, though not all smokers develop lung cancer. Sex differences in the metabolism of tobacco smoke predispose females to developing COPD and accumulating damage from oxidative stress that poses a risk for the development of lung cancer. Dysregulation of the lung microenvironment and microbiome contributes to chronic inflammation, which is observed in COPD and known to facilitate cancer initiation in various tumor types. Further, there is a need to better characterize and identify the proportion of individuals with COPD who are at a high risk for developing lung cancer. We evaluate possible novel and individualized screening strategies, including biomarkers identified in genetic studies and exhaled breath condensate analysis. We also discuss the use of corticosteroids and statins as chemopreventive agents to prevent lung cancer. It is crucial that we optimize the current methods for the early detection and management of lung cancer and COPD in order to improve the health outcomes for a large affected population.

## 1. Introduction

**Figure 1:**
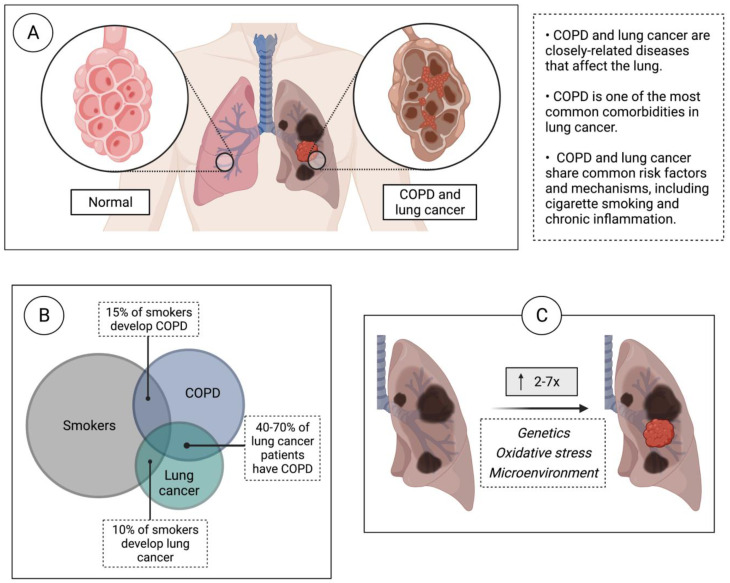
COPD confers a greater risk for lung cancer development. (**A**) COPD and lung cancer are closely linked diseases that affect the lung. (**B**) There is a large overlap between COPD and lung cancer in smokers. While a subset of smokers develop COPD or lung cancer, a significant proportion (40–70%) of lung cancer patients have COPD. (**C**) COPD confers a 2-7x greater risk of developing lung cancer, independent of smoking history. Possible shared mechanisms that may contribute to this process are genetics, oxidative stress, and microenvironmental factors.

Lung cancer is the leading cause of cancer-related mortality worldwide and accounts for almost 25% of all cancer fatalities. Despite recent advances in diagnosis and treatment, the five-year survival rate remains poor at 22%. Lung cancer is divided broadly into the categories of small cell lung cancer (SCLC) and non-small cell lung cancer (NSCLC), which show variable disease etiology, risk factors, and genomic variants associated with the onset of disease [1,2]. Largely, it has been shown that squamous cell carcinoma (SCC), a subtype of NSCLC, has a greater association with COPD (e.g., risk, decreased overall survival, severity), as well as an increased risk of SCLC with the presence of COPD independent of smoking status [3,4,5,6,7]. However, no biological link has been identified. Tobacco smoking is a major cause of lung cancer, and other smoking-related conditions such as cardiovascular diseases and chronic obstructive pulmonary disease (COPD) often present as comorbidities (Figure 1). In all, 40-70% of individuals with lung cancer show evidence of airflow obstruction, indicative of COPD, which is commonly measured by the forced expired volume in one second (FEV1) (Figure 1B) [8]. Patients with COPD experience a continual inflammation of the lung tissues, which has been shown to increase the risk of developing lung cancer two to seven fold, irrespective of smoking history [9,10,11]. The mechanisms accounting for this elevated risk are not understood; thus, deciphering such mechanisms could yield targets for early intervention to prevent COPD-damaged airways from progressing to cancer [12]. Additionally, chronic inflammation is known to increase the metastatic potential of cancer [13,14,15], and one study has linked a pathway active in COPD to increased migration in the NSCLC cell lines [16]. There is also some evidence that co-existing COPD can promote the metastasis of other primary tumors to the lungs [17,18,19]. Furthermore, COPD in never-smokers was recently shown to be an independent predictor of lung cancer incidence [20]. Worsening airflow limitation is correlated with increasing lung cancer incidence, signifying that there is a linear relationship between increasing COPD severity and cancer risk [21]. Lung cancer patients with COPD, compared to those without COPD, show significantly worse clinical and surgical outcomes, indicating an urgent need for improved screening and early treatment options for lung cancer in the COPD population. Considering the prevalence of COPD and the mortality rate of lung cancer, it is crucial to identify early intervention strategies to detect and prevent lung cancer onset in this high-risk patient group. We review potential mechanisms associated with the transition between COPD and lung cancer including genetic predisposition, epigenetic changes, the alteration of non-coding RNAs (ncRNAs), oxidative stress-induced pathogenesis, immune microenvironment and microbiome dysregulation, and sex differences in the susceptibility to lung cancer and COPD [22]. We then highlight the application of this knowledge in optimizing lung cancer screening and prevention in the context of COPD to improve patient outcomes.

## 2. Genetic Mechanisms of Lung Cancer Development in Individuals with COPD

**Figure 2:**
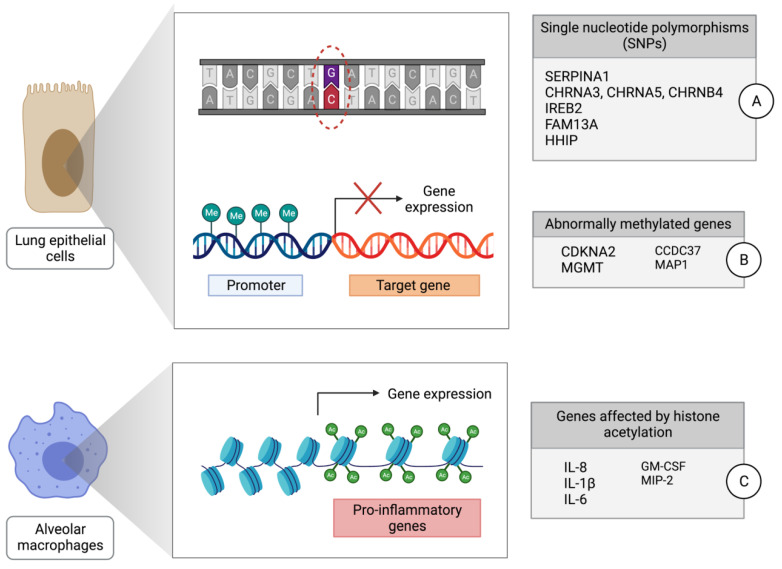
Shared genetic and epigenetic mechanisms in COPD and lung cancer development. (**A**) Single nucleotide polymorphisms (SNPs) carries increased risk for the development of both COPD and lung cancer. (**B**) Abnormal promoter methylation decreases the expression of certain tumor suppressor and immune genes. (**C**) Histone acetylation of pro-inflammatory genes in alveolar macrophages leads to increased expression of pro-inflammatory mediators.

Genetic alterations as well as the deregulated expression of protein-coding and non-coding RNA genes have been extensively reported for the subtypes of lung cancer [23,24,25]. While the genetic contribution to COPD is not fully understood [26], studies have linked the co-morbidity of lung cancer and COPD [12]. For example, two percent of COPD patients have an alpha-1 antitrypsin (A1AT) deficiency that results in unopposed proteolytic activity by neutrophil elastase (NE), which has been linked to increased lung cancer progression and metastasis [27,28]. Genome-wide association studies (GWAS) have also identified several genetic variants that might increase the susceptibility to developing both COPD and lung cancer (Figure 2A); however, the estimated heritability of both remains low [29,30]. Polygenic risk scores (PRSs) show a high association between genotype and the onset and severity of developing COPD, both in European and in non-European populations [31], with multi-ancestry PRS performing better than ancestry-specific ones [32]. Other variants include certain shared single nucleotide polymorphisms (SNPs) that also appear to increase the risk of both COPD and lung cancer development, particularly those found on chromosome 12q25 [33] and chromosome 6 [34]. Another cluster of genes, specifically the *CHRNA5*-*CHRNA3*-*CHRNB4* loci on chromosome 15, encode the nicotinic acetylcholine receptor (nAChR) subunits that are expressed on lung epithelial cells, and variants in this region have been associated with a heightened COPD and lung cancer risk [35]. A recent prospective cohort study found that the CHRNA5 AA genotype variant was independently associated not only with an increased risk of developing COPD and lung cancer, but also with increased smoking exposure, referred to as the ‘triple whammy effect’ [36]. Telomere shortening has also been linked to lung cancer subtypes and COPD [37], with the *rs2736100* variant of the telomerase reverse transcriptase (*TERT*) having been shown to be associated with an increased risk of COPD [38]. The *rs560191* allele variant of tumor protein p53-binding protein 1 (TP53BP1) may increase the lung cancer risk depending on which polymorphism of the *TERT* allele is present, indicating a potential combinatorial effect [39]. Other SNPs that have been linked to COPD and lung cancer susceptibility include those found in *IREB2*, *FAM13A*, and *HHIP* [40,41]; however, the majority of these seem to only confer a small additive magnitude of effect. These shared genes imply shared pathways, and early interventions at the onset of COPD could depress the progression of COPD towards lung cancer.

## 3. Epigenetic Mechanisms of Lung Cancer Development in Individuals with COPD

Epigenetic mechanisms have also linked COPD and lung cancer development, and specific patterns of DNA methylation have been identified in both COPD and lung cancer patients. Genome-wide alterations in the methylation status of genes can be induced by tobacco smoking and may affect genes involved in cell-cycle regulation, airway remodelling, and wound healing, to name a few [42,43]. These abnormal patterns of promoter methylation in COPD can also alter the expression of tumor suppressor or immune genes to promote carcinogenesis (Figure 2B), with global hypomethylation observed in whole blood samples and modulated levels found in sputum samples by cell type [44,45]. Hypermethylation leading to decreased expression levels of *CDKN2A*, *MGMT*, *CCDC37*, and *MAP1B* genes have been found in individuals with both COPD and lung cancer [46,47]. Interestingly, the increased levels of methylation found in *IL-12Rb2* and *Wif-1* genes in COPD patients were reported to contribute to COPD-related lung cancer [48]. Furthermore, a hypermethylation and underexpression of the tumour suppressor gene *PTEN* was seen in COPD patients [30]. *PTEN* is commonly found to be mutated in cancers, especially in smokers. If COPD patients have changes in the methylation status of tumour suppressor genes that lead to their decreased expression, and subsequently acquire mutations in such genes, or vice versa, this could lead to the biallelic inactivation of these genes and thus promote lung tumorigenesis.

Hypomethylated cytosine-phosphate-guanine (CpG) sites within certain genes have been significantly associated with the presence and severity of COPD [49]. Gene ontology analysis based on these CpGs has identified several genes, especially in the gene coding for alpha1-antitrypsin (*SERPINA1*), involved in immune and inflammatory system pathways which may further contribute to tumor proliferation, migration, and invasion, where *SERPINA1* hypomethylation has been linked to COPD and decreased lung function [49]. A recent study reported that, in male patients with COPD, higher levels of serum *SERPINA1* methylation are associated with the development of lung cancer [50]. It has also been demonstrated that IL-1β, an important inflammatory biomarker of COPD previously associated with lung cancer, is positively correlated with the *SERPINA1* methylation levels. Interactions between *SERPINA1* and IL-1β and their role in disease is not yet fully understood. Aberrant DNA methylation has been recently reviewed in lung cancer [51], but it may also present therapeutically targetable drivers and biomarkers of lung cancer in patients with COPD.

The dysregulation of histone modifications can also contribute to COPD and lung cancer development through the modification of pro-inflammatory gene expression (Figure 2C). In alveolar macrophages, the inflammation-induced histone acetylation of *IL-8*, *IL-1b*, *IL-6*, *GM-CSF*, and *MIP-2* genes results in an elevated expression of these inflammatory mediators [52]. Increased histone acetyltransferase (HAT) and decreased histone deacetylase (HDAC) activity are also characteristic, contributing to an inflammatory environment that promotes cancer growth. Furthermore, as environmental influences affect the onset of COPD substantially, the epigenetic profiles of susceptible people might be prone to change dynamically over time.

## 4. Alterations of Non-coding RNAs in COPD and Lung Cancer

Non-coding RNAs (ncRNAs) are a heterogeneous class of RNAs that are not translated into proteins and are involved in a wide variety of biological functions, including the regulation of gene expression, RNA processing, and translation [53]. They are divided into two broad categories: small ncRNAs (sncRNAs; <200 nucleotides) and long ncRNAs (lncRNAs; >200 nucleotides) [54,55]. There are also multiple classes of small ncRNAs, including microRNAs (miRNAs), PIWI-interacting RNAs (piRNAs), small interfering RNAs (siRNAs), and small nucleolar RNAs (snoRNAs). NcRNAs have also been frequently implicated in cancer and appear to show specific patterns of expression in COPD compared to non-COPD patients. Some of these ncRNAs have also been previously linked to lung cancer.

### 4.1. MicroRNAs

Currently, the majority of the studies on the role of ncRNAs in both COPD and lung cancer has focused on miRNAs. The dysregulated expression of miRNAs in COPD patients contributes to known important mechanisms in the pathogenesis of lung cancer, such as airway inflammation [56], angiogenesis [57], immune response [58], autophagy [59], oxidative stress [60], and cellular senescence [61], suggesting that miRNA alterations may precede the development of lung cancer. From a clinical perspective, circulating miRNAs in the serum have shown promise as clinical markers for lung inflammatory disease and response to treatment [62]. In COPD, miR-218-5p has been previously reported to be downregulated in biopsies from patients with COPD and smokers, potentially indicating a protective role in the pathogenesis of COPD and spurring an ongoing clinical trial evaluating circulating miRNAs in smokers and non-smokers with COPD [63,64]. In addition, several studies have confirmed that alterations in the miRNA expression levels could increase the risk of developing cancer in COPD patients. For example, the downregulation of cancer-associated miRNAs, such as miR-548D-5p, miR-4695-3p, miR-517a-3p, miR-320E, miR-548ay-5p, miR-320c, and miR-519D-3p, was observed in COPD patients, and suppression of these miRNA was associated with an increased lung cancer risk [65]. Another study evaluating bronchoalveolar lavage samples showed the miRNAs (miR-15b, miR-425, and miR-486-3p) commonly expressed by patients with COPD and those with adenocarcinoma, as well as by those with both COPD and adenocarcinoma [66]. An investigation by Fathinavid et al. observed four miRNAs (miR-15b, miR-106a, miR-17, miR-103, and miR-107) involved in common pathways in both COPD and lung cancer [67]. According to pathway analyses, these miRNAs might be involved in the VEGF signaling, ERBB signaling, WNT signaling, and TGF beta signaling pathways; however, further validation via in vitro experiments will be required [67]. Similarly, the comparison of the miRNA expression profiles of lung samples from lung cancer patients with and without COPD identified four miRNAs (miR-21, miR-200b, miR-210, miR-let7c) that demonstrated greater expression in lung cancer patients with COPD than in those without. Furthermore, these miRNAs appeared to regulate target genes related to lung carcinogenesis [68]. Several studies have shown that miR-21 is an oncogenic miRNA (oncomiR) involved in the growth, metastasis, and apoptosis of NSCLC cells through its control of various target molecules and signaling pathways [69,70,71,72]. In COPD, an increase in miR-21 expression in airway epithelium and lung macrophages is implicated in airway inflammation and fibrosis, and reduces lung function [73]. One recently reported method of modulating miRNA expression is the ketogenic diet, which was shown to normalize the levels of miRNAs controlling the metabolic network in obesity, showcasing the therapeutic potential of miRNAs in inflammatory disease [74]. Overall, this seems to indicate a potential role for miRNAs as biomarkers for COPD-associated lung cancer. Additionally, it would be rational to explore whether the turnover of specific miRNAs may be a new therapeutic strategy for these patients.

### 4.2. Long Non-Coding RNAs

Studies have also revealed an association between lncRNAs and the susceptibility to both COPD and lung cancer [75,76]. Evidence shows that Metastasis-associated lung adenocarcinoma transcript 1 (*MALAT1*, also called nuclear enriched abundant transcript 2 or *NEAT2*), an lncRNA highly abundant in lung tissues, is implicated in the pathogenesis of chronic lung diseases, including both COPD and lung cancer [76]. There is evidence that, in NSCLC patients, *MALAT1* can accelerate proliferation, migration, and invasion and impede apoptosis through regulating the miR-185-5p/*MDM4* axis, while promoting cell carcinogenesis via the miR-515-5p/*EEF2* axis [77,78]. In COPD, a high *MALAT1* expression mediated by a hypomethylated promoter elevates cyclooxygenase 2 (*COX2*) expression via inhibiting miR-146a, which can affect the pulmonary functions in COPD patients [79]. Another investigation demonstrated that a high expression of *MALAT1* can be used to predict the disease progression and acute exacerbation risk in COPD patients [80]. *MALAT1* has a well-established role in lung cancer progression and carcinogenesis [77,78,81], but further research will be required to uncover common links between its mechanisms of action in COPD and lung cancer.

The lncRNAs NNT Antisense RNA 1 (*NNT-AS1*) and growth arrest-specific 5 (*GAS5*) can also contribute to the increased risk for lung cancer in COPD patients. In comparison with NSCLC patients without COPD, the level of *NNT-AS1* expression was enhanced in lung tissues from patients with COPD [82]. In vitro, *NNT-AS1* promotes proliferation, apoptosis, inflammation, and airway remodeling by regulating the expression of the target gene *FBXO11* via sponging miR-582-5p [82]. In COPD, *GAS5* promotes pyroptosis (a well-documented process in cancer [83]) by functioning as competing endogenous RNAs to regulate the miR-223-3p/*NLRP3* axis [84].

### 4.3. Small Nucleolar RNAs

Although emerging evidence has also demonstrated that snoRNAs are essential factors in lung cancer onset and dissemination, their roles in lung cancer susceptibility in COPD patients remain unknown [85]. For instance, SNORD66 (Small Nucleolar RNA, C/D Box 66) were found at elevated levels in plasma samples in both COPD and NSCLC patients when compared with healthy individuals [86]. The area under the receiver operating curve (AUC) for this snoRNA was 0.81 (lung cancer patients versus healthy controls) and 0.79 (lung cancer patients versus COPD patients), suggesting that snoRNAs provide potential markers for differentiating between lung cancer and COPD. Further studies will be required to determine the role of lncRNAs and snoRNAs in lung cancer susceptibility in COPD patients to evaluate their contribution to pathogenesis and potential for use as biomarkers for lung cancer in the COPD population.

## 5. Oxidative Stress Contributes to Pathogenesis in COPD and Lung Cancer

**Figure 3:**
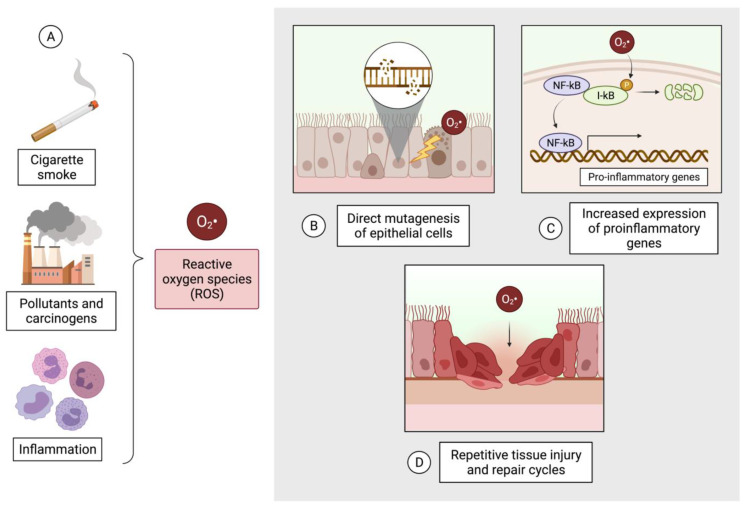
Oxidative stress is a common underlying mechanism in the pathogenesis of COPD and lung cancer. (**A**) It is generated in the presence of inflammation, tobacco smoke, pollutants, and carcinogens. (**B**) Reactive oxygen species (ROS) directly causes DNA damage in lung epithelial cells. (**C**) ROS increases the expression of pro-inflammatory genes in a NK-kB-dependent manner. (**D**) Repeated cycles of tissue injury and repair occur due to damage from ROS, which involves the process of epithelial-mesenchymal transition.

Oxidative stress is a major mechanism underlying the pathogenesis of COPD and lung cancer, and it occurs when an exposure to reactive oxygen species (ROS) overwhelms the host’s antioxidant defenses. ROS are generated by tobacco smoke, a risk factor for both diseases, but can also be produced by exposure to other pollutants and carcinogens (Figure 3). ROS and reactive nitrogen species (RNS) are both generated in the presence of airway inflammation. Free radicals from ROS and RNS can oxidize and damage DNA and can post-translationally modify DNA repair proteins [87]. This is evident in the lungs of COPD patients, which have increased oxidative DNA damage and impaired DNA repair mechanisms compared to non-COPD smokers and non-smokers (Figure 3A) [88,89], resulting in an increased number of double strand breaks at abasic sites and a reduced expression of DNA repair enzymes. ROS and RNS can also activate signaling molecules such as HIF1, the master regulator of angiogenesis and an important factor for tumour progression [90]. Additionally, the by-products of ROS and inflammation have been shown to inactivate PTEN, a commonly-altered tumour suppressor gene in lung cancer, through the formation of an intramolecular disulfide bond [91,92]. DNA damage, combined with impaired DNA repair mechanisms in COPD, can predispose lung epithelial cells to acquiring somatic mutations and progressing to malignancy.

ROS also contributes to the cycle of inflammation, immune response, and oxidative stress by activating NF-kB, a transcription factor that promotes cellular proliferation, suppresses apoptosis, and activates the epithelial-mesenchymal transition (EMT; Figure 3B) [93,94]. The lung epithelium of COPD patients and smokers has upregulated mesenchymal markers, such as *α*-SMA, vimentin, and collagen type 1, and downregulated epithelial markers (e.g., ZO-1, E-cadherin) compared to that of non-COPD non-smokers, likely due to cell turnover from repeated cycles of tissue injury and repair due to inflammation and oxidative stress (Figure 3C) [95,96]. Moreover, tobacco smoke extract lowered the levels of zonula occludens-1 (ZO-1), an epithelial marker involved in tight junction structure, both in a normal lung cell line and in the airways of mice [97]. However, in situ analysis did not detect ZO-1 downregulation in the lung tissue of smokers [96]. This reduction in epithelial attributes seen with airway repair can impair epithelial barrier function, and has been correlated with subepithelial fibrosis and airway obstruction, key characteristics of COPD pathogenesis [96]. This also has implications for cancer, as increased airway permeability and EMT can promote the local invasion of cancer cells and thus the distant metastasis of cancer cells through the blood vessels. Therefore, the presence of COPD could potentially contribute to carcinogenesis and worse outcomes in lung cancer. Collectively, the effects of oxidative stress can induce multiple pre-malignant fields of injury in the respiratory system that are at a higher risk of cancer formation.

## 6. Lung Immune Microenvironment Dysregulation Contributes to Both COPD and Lung Cancer Progression

**Figure 4:**
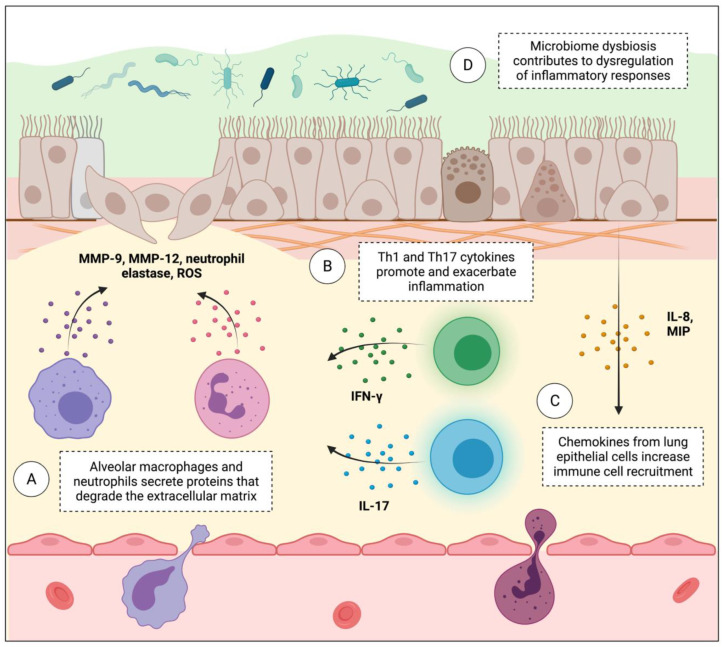
Features of immune and microenvironment dysregulation in COPD that contribute to cancer risk. (**A**) A Th1 T cell polarization is characteristic of COPD. Th1 cells secrete pro-inflammatory cytokines that activate neighbouring immune cells and exacerbate the inflammatory response. (**B**) Macrophages and neutrophils release ROS, matrix metalloproteinases (MMPs), and neutrophil elastase (NE), which contributes to tissue injury and extracellular matrix (ECM) remodeling. (**C**) IL-17 secreted by Th17 T cells and chemokines released by the lung epithelium promote increased neutrophil and macrophage recruitment. (**D**) Dysbiosis of the lung microbiome is evident in both COPD and lung cancer and may play a role in promoting immune dysregulation.

The lung immune microenvironment has been well-researched in the field of COPD and lung cancer, and its dysregulation is known to play a role in the formation of a tumour-promoting niche [98,99]. In COPD, the immune microenvironment encompasses the states of both chronic airway inflammation and emphysema, and the progression of both phenotypes has been linked to inhaled irritants, oxidative stress, and immune cell deregulation. Chronic inflammation has been established as a significant risk factor for the development of various cancer types. Individuals with COPD have a higher degree of airway and systemic inflammation compared to healthy individuals, and studies have shown that this inflammation persists even after the cessation of smoking [100]. Chronic inflammation is common between COPD and lung cancer, and alterations of inflammatory mediators and of immune cell composition may play a major role in pathogenesis [101,102]. The increased infiltration of neutrophils, alveolar macrophages, CD4+ and CD8+ T cells, and B cells is also associated with the severity and stage of COPD. Inflammation in COPD is characterized by a Th1 T cell phenotype, which can initiate or exacerbate lung inflammation by secreting IFN*γ*, IL-2, and TNF*α* (Figure 4A). Neutrophils and alveolar macrophages are potent sources of oxidative stress and have various functions that can contribute to an increased risk of lung cancer (Figure 4B).

Neutrophilic inflammation in COPD is associated with increased disease severity and exacerbations, and, in lung cancer, a high neutrophil to lymphocyte ratio (NLR) is associated with poor prognosis and survival [103]. Both neutrophils and macrophages can contribute to exacerbating emphysema in COPD, and they also support cancer growth and metastasis by secreting NE and other metalloproteinases (MMPs). In particular, MMP-1 and MMP-9 have both been linked to the development of emphysema and lung cancer. Higher amounts of IL-17A, primarily secreted by Th17 cells, are also found in individuals with COPD, compared to healthy smokers and non-smokers (Figure 4C) [104]. IL-17A is involved in the recruitment of neutrophils and macrophages to the lung, and increased amounts are associated with COPD severity. Chronic NF-kB signalling may also provide an immunosuppressive tumor microenvironment in the lung favourable for the development of both COPD and lung cancer [105]. Further contributing to the oncogenic potential of chronic inflammation is the fact that inflammation recruits myeloid-derived suppressor cells (MDSCs), which down-regulate immune surveillance and antitumor immunity, thus facilitating tumor growth [106]. Overall, having COPD results in changes to the immune cells that infiltrate the lung, and their presence can alter components of the microenvironment, such as the ECM, and recruit more immune cells that are able to create a tumour-promoting microenvironment.

## 7. The Lung Microbiome in COPD and Lung Cancer

Although once thought to be sterile, the airways of the lungs are now known to host a distinctive microbiome. The contribution of microbes to the microenvironment in disease contexts is significant; microbial dysbiosis has been implicated in cancer initiation through the promotion of inflammation and the generation of ROS, and by signalling epithelial cells directly to activate oncogenes (Figure 4D) [107]. While normal lungs are hosts to a healthy microbiome, there is evidence to suggest links between an abnormal lung microbiome composition, COPD, and the inception of lung cancer.

Significant differences have been found between the microbial communities of healthy and diseased airways in COPD and lung cancer. Alpha diversity (the number of taxa and the evenness of their distribution in samples) tends to be higher in normal lung tissue than in lung cancer tissue [108]. The bacterial composition of diseased lungs can shift from the phylum *Bacteroidetes* towards the class Gammaproteobacteria, many members of which are gram-negative pathogens [109]. Comparing patients with COPD, lung cancer, or both, the proportion of gram-negative bacteria has been found to be significantly higher in those with both diseases [110]. One study found that patients with severe COPD have larger populations of species from the *Lactobacillus* genus than those with less severe or absent COPD [111], and another study found that a high *Lactobacillus* population was a good predictor of lung cancer [112]. Other studies have found increases in the genera *Veillonella*, *Streptococcus*, and *Granulicatella*, the latter of which particularly increased in late-stage cancer [113,114]. Bacteria of the genus *Acidovorax* were found to be abundant in the lung tissue of smokers with squamous cell carcinoma and *TP53* mutations [115]. A meta-analysis has described a significant association between tuberculosis and the onset of lung adenocarcinoma, independent of smoking history, environmental smoke exposure, or time of tuberculosis diagnosis [116]. These distinctive microbiome compositional changes suggest that bacteria are sensitive to microenvironmental changes in the lung, which may be common between COPD and lung cancer and may even influence them.

Changes in the microenvironment of the airways during disease progression could allow pathogenic microbiota to overtake commensal species. These pathogenic species may in turn exacerbate conditions that are conducive to their survival. Dysbiosis, or the pathological imbalance of bacterial species, is thought to increase the amount of DNA damage-associated ROS in the lungs [108]. The by-products of inflammation may provide some *Gammaproteobacteria* with terminal electron acceptors for anaerobic respiration, allowing them to outcompete fermenting bacteria [109]. Some *Gammaproteobacteria* also produce inflammation-promoting molecules, which could create a positive feedback loop allowing them to thrive. Proportionally abundant species found in the lungs of patients with COPD and lung cancer were associated with oxidative stress, amino acid metabolism, and glycolipid metabolism, which could influence cancer development [110]. Bacterial metabolic by-products, such as acetaldehyde and deoxycholic acid, are noted carcinogens [108]. Bacteria can also activate toll-like receptors (TLRs), which drive the cellular pathways associated with proliferation and survival. Tuberculosis is known to increase TNF-α expression, which promotes inflammation and can thereby cause genetic damage [108].

There are limitations to the studies of COPD and lung cancer-associated microbiota, which include different sample collection methods between studies, a sampling bias towards those with lung disease, and the inherent difficulty in determining whether population shifts at a diseased site are opportunistic or causative [108,113,117]. However, the utility of the microbiome as a marker of disease is an attractive strategy and is currently being explored. In fact, a recent study proposed that a classifier based on the lung microbiome profile could be used to detect lung cancer in its early stages [112]. While the exact pathogenic role of bacteria in the airways has not yet been fully elucidated, there are compositional changes in the tissue-resident microbiome which can be used to indicate the presence of lung disease, and potentially to predict cancer development [112].

## 8. Sex Differences in COPD and Lung Cancer Risk

Female sex is a known risk factor for the development of COPD in smokers; however, sex differences in lung cancer risk are controversial, and sex differences in non-smokers for both COPD and lung cancer risks are topics for further research. It has been shown in large epidemiological studies that women who smoke are 50% more likely to develop COPD than men [118] and are two to three times more likely to die of COPD than men for any given pack-year of smoking [119]. It has also been widely reported that women are more susceptible to the harmful effects of smoking and are at a greater risk of developing COPD [120]. One explanation is that the smaller lung size in women means the damage caused by oxidative stress is more pronounced than in men with a comparable smoking history [120]. Another is sex differences in tobacco metabolism: women have an increased activity of certain cytochrome P450 enzymes in the liver, such as CYP1A1 and CYP1B1, which activate certain components of tobacco smoke to produce ROS [121]. This increased CYP expression in females is due in part to the action of estrogen in inducing CYP enzyme-related pathways [122]. For example, a study of smokers with lung cancer demonstrated an increased expression of CYP1A1 in females and a corresponding increase in DNA adducts even in non-tumor lung tissue [123]. Additionally, work performed in animal studies demonstrated that the injection of naphthalene (a component of tobacco smoke) into female mice caused greater airway injury, spurred by an increased CYP enzyme expression and the production of a metabolites that initiated a more extreme inflammatory reaction in the airways and generated more ROS than in males [124]. In non-smokers, indoor and outdoor air pollution exposure is also a significant risk factor for the development COPD in women, who are more commonly exposed to biomass smoke [125,126].

The evidence that female smokers are at a higher risk for developing lung cancer relative to male smokers has been inconclusive. On one hand, increased susceptibility to the harmful effects of smoking via the increased expression of cytochrome P450s, mediated partially through estrogen signalling, has been reported [127], and early case-control studies demonstrated an increased risk of developing lung cancer in female smokers compared to in males [128]. Epidemiological studies also found an increased odds ratio for the development of lung cancer in female smokers relative to male smokers [129,130]. In contrast, it has also been reported that there is no difference in the risk of lung cancer once smoking history is accounted for [131], or that there is a potential slight increase in risk in male smokers compared to female smokers [132]. A general trend in the last 50 years is that women have shown an increased risk of cancer relative to men, but this has been attributed to rising rates of adenocarcinoma in younger women and in never-smokers [133]. Risk factors other than smoking that may be unique for women include exogenous factors such as exposure to radiation from the treatment of prior cancers, such as breast cancer, and biomass exposure from indoor cooking fumes, and endogenous factors such as exposure to hormone replacement therapy and the previously discussed increase in CYP450 enzyme expression, leading to increased ROS production [134]. Furthermore, for the same decrease in FEV1, female smokers are twice as likely to develop lung cancer as men are [21], reinforcing a role for lung disease like COPD in the pathogenesis of lung cancer.

There is evidence of an increased risk of COPD in female smokers, but an increased risk of lung cancer in this population has not been proven, despite the known mechanisms of female-specific increased DNA damage from tobacco smoke. The factors accounting for the rise in lung cancer rates in younger females and never-smokers, including potential associations with COPD, have the potential to better inform screening guidelines for these populations.

## 9. Implications for Lung Cancer Screening and Prevention

**Figure 5:**
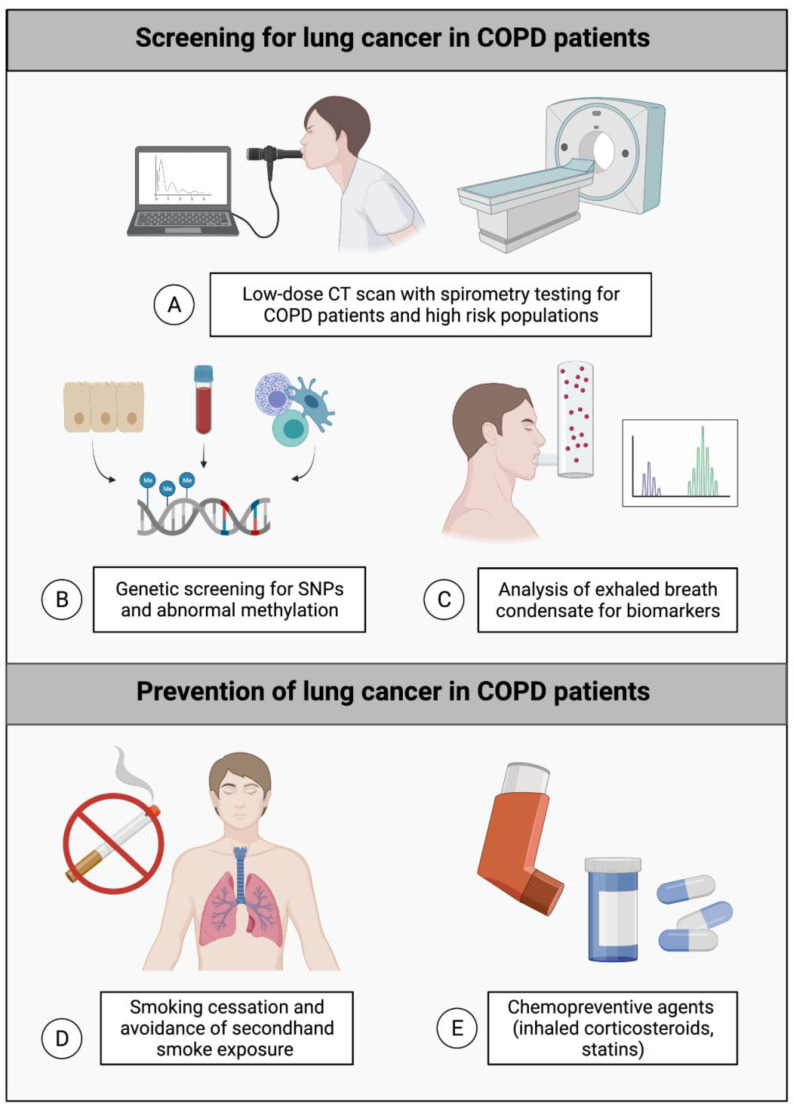
Potential strategies for lung cancer screening and prevention in the COPD population. (**A**) Offering low-dose CT scans combined with spirometry testing to all individuals with COPD and those at high risk for COPD may improve early lung cancer detection. (**B**) Using genetic screening to detect SNPs and abnormal methylation patterns, or (**C**) analysis of biomarkers of oxidative stress and inflammation in exhaled breath condensate (EBC) may be used to determine which COPD patients are at a higher risk of lung cancer development. (**D**) Smoking cessation and avoidance of second-hand smoke exposure is currently the best method of cancer prevention in COPD. (**E**) Some chemopreventive agents, including inhaled corticosteroids (ICS) and statins, have shown limited efficacy in preventing lung cancer in COPD.

The 2013 guidelines from the United States Preventive Services Taskforce (USPSTF) recommends annual lung cancer screening with low-dose computed tomography (CT) for adults 55–80 years old with a 30 pack-year history. However, several studies have shown that these guidelines lack sensitivity and specificity. Approximately 60% of individuals with lung cancer do not meet the screening criteria, and 96% of the nodules identified were false positives [135]. Females and non-smokers are particularly underrepresented, and risk factors other than smoking history are not accounted for—50–80% of women diagnosed with lung cancer do not meet the USPSTF screening criteria [134]. Although the USPSTF guidelines were updated in 2021 to expand eligibility since these statistics were published, lowering the minimum age to 50 and the pack-year history to 20 [136], a substantial and rising proportion of the young female population will continue to be excluded. Therefore, there is a need to better identify unique risk factors for underdiagnosed populations in screening paradigms beyond simple age and smoking status. For example, COPD is vastly underdiagnosed in the population, and including spirometry testing during lung cancer screenings may lead to the earlier detection of undiagnosed COPD, thus expanding the window for intervention to prevent cancer development (Figure 5A). As up to 70% of individuals with lung cancer have COPD, offering screening to current and former smokers with COPD regardless of age or pack-years can theoretically increase early lung cancer detection, but this is limited by the potential for over-diagnosis. Alternative methods to CT scans using biomarker identification may be used to screen for lung cancer in the future. Genetic tools, including the identification of methylation signatures and GWAS, have been used to assess the risk of lung cancer with measured success (Figure 5B) [137]. For instance, the hypermethylation of Prostaglandin E Receptor 4 (*PTGER4*) in the plasma was shown to distinguish patients with lung cancer from those with COPD or benign lung lesions [138]. Further, markers associated with oxidative stress and inflammation can be measured in exhaled breath condensate (EBC), and certain biomarkers have already been characterized for both COPD and lung cancer (Figure 5C) [139]. Another application for biomarkers is in prognosticating: for example, a signature of five genes was discovered that is overexpressed in lung cancer with comorbid COPD and can be used to predict the survival of the lung cancer–COPD population [140]. Stratifying patients using prognostic biomarkers may facilitate clinical decisions to improve patient outcomes.

Currently, the most well-established ways of preventing lung cancer in patients with COPD are smoking cessation and limiting exposure to second-hand smoke (Figure 5D). Some chemopreventive agents including inhaled corticosteroids (ICS), which are commonly prescribed to COPD patients, and statins have been associated with reduced inflammation and thus with a decreased cancer risk (Figure 5E). However, the evidence for their effectiveness in lung cancer prevention is controversial, as several observational studies have demonstrated a dose-dependent reduction in lung cancer risk with ICS or statin use [141,142], while systematic reviews and meta-analyses found no significant benefit [143,144]. Statins have also been considered as a treatment for lung cancer, but, despite multiple suggested mechanisms of action, the clinical evidence of efficacy is lacking [145]. The inhibition of interleukin 1β, a mediator of chronic inflammation in the tumour microenvironment that has been indirectly associated with COPD, with canakinumab in patients with atherosclerosis was incidentally demonstrated in a randomized controlled trial to reduce lung cancer incidence and mortality compared to placebo [146,147]. Though canakinumab reduced lung cancer incidence, it had no effect on COPD incidence, suggesting a more prevalent role for interleukin 1β in the progression of lung cancer than in COPD and highlighting the importance of studying the differences between COPD vs. non-COPD-related lung cancer. A recent retrospective cohort study reported that low-dose aspirin exposure in patients with COPD reduced the risk of lung cancer development by 25%, although aspirin use was accompanied by an increased risk of the bleeding disorder hemoptysis [148]. A previous meta-analysis reported no association between lung cancer risk and aspirin use [149], and a retrospective cohort study found that aspirin only decreased the lung cancer risk in combination with metformin and statins [150]. This could potentially indicate a chemopreventive role for aspirin specifically in the context of patients with COPD, though further research will be required to validate this finding. Further revision of the screening guidelines to better capture underdiagnosed cohorts (including females and non-smokers), incorporating novel biomarkers, and delineating the mechanisms linking COPD with lung cancer, will be essential for developing effective prevention strategies in the future.

## 10. Conclusions

Despite the notably poor outcomes in individuals with both COPD and lung cancer, there are few available strategies for preventing the development of lung cancer in the context of COPD.

Determining genetic variants and epigenetic alterations helps to stratify the COPD population according to their risk of developing lung cancer. Delineating the temporal changes in the tissue immune microenvironment and the microbiome of the pre-malignant lung is also a key step in developing treatment strategies to block progression. Investigating these factors with attention to sex-specific differences will be essential, as an increased susceptibility to COPD in females has been reported. A better understanding of the biological factors that drive the progression from COPD to lung cancer, in addition to the development of screening tools to effectively detect which individuals with COPD are most likely to develop lung cancer, has the potential to identify and improve the outcomes for a very large at-risk population.

## Data Availability

Not applicable.

## Abbreviations

COPD	Chronic Obstructive Pulmonary Disease
CT	Computed tomography
ECM	Extracellular matrix
EMT	Epithelial-mesenchymal transition
FEV	Forced expiratory volume
GWAS	Genome-wide association studies
ICS	Inhaled corticosteroids
lncRNA	Long non-coding RNA
miRNA	MicroRNA
MMP	Matrix metalloproteinase
NE	Neutrophil elastase
ncRNA	Non-coding RNA
NLR	Neutrophil to lymphocyte ratio
NSCLC	Non-small cell lung cancer
PRS	Polygenic risk score
ROS	Reactive oxygen species
SCLC	Small cell lung cancer
SNP	Single nucleotide polymorphism
USPSTF	United States Preventive Services Task Force
